# Effects of a WeChat-based PERMA model positive psychological intervention on Chinese women after termination of pregnancy: a randomized controlled trial

**DOI:** 10.3389/fpsyg.2026.1823918

**Published:** 2026-07-20

**Authors:** Shaofang Ye, Xiumei Xiong, Zhumei Lin, Meirong Wen, Lingna Huang, Yinzhi Li, Chenyin Liu

**Affiliations:** Department of Gynecology, Fujian Maternity and Child Health Hospital, Fuzhou, Fujian, China

**Keywords:** grief, PERMA model, positive psychological intervention, post-traumatic growth, termination of pregnancy, WeChat group

## Abstract

**Objective:**

To explore the effects of WeChat-based PERMA (Positive Emotion, Engagement, Relationships, Meaning, and Accomplishment) positive psychological intervention on psychological adaptation during puerperium among women who underwent termination of pregnancy (TOP), with a focus on evaluating its efficacy in alleviating grief, promoting post-traumatic growth, and enhancing subjective wellbeing.

**Methods:**

A randomized controlled trial was conducted with 118 women who experienced TOP at a tertiary Grade A hospital in Fujian province, China. Participants were randomly allocated to an intervention group (*n* = 59) receiving 4-week WeChat-based PERMA model intervention alongside routine postpartum care, or a control group (*n* = 59) receiving routine care only. Repeated measures were used to compare score changes in the Perinatal Grief Scale, Chinese version of Post-Traumatic Growth Inventory, and Index of Wellbeing at baseline, 1-month post-intervention, and 3-month post-intervention.

**Results:**

The intervention group demonstrated significantly greater reductions in grief at both follow-ups (both *p* < 0.001, Cohen's *d* > 1.3), as well as improvements in post-traumatic growth and subjective wellbeing, with effect sizes ranging from moderate to large. All intervention effects increased over time.

**Conclusions:**

The WeChat-based PERMA intervention significantly reduces grief intensity (to a mean score well below the established threshold for high-level grief), promotes post-traumatic growth, and enhances wellbeing in women after TOP. This nurse-led digital program offers a scalable, low-resource support option for bereavement care during the puerperium.

**Clinical Trial Registration:**

https://www.chictr.org.cn/showproj.html?proj=226100, identifier: ChiCTR2400087627.

## 1 Introduction

Termination of pregnancy (TOP) for severe fetal indications represents a significant global health issue. For the purpose of this study, TOP is defined broadly to include not only termination for fetal anomaly but also the medical management of intrauterine fetal death and missed abortion at ≥12 weeks of gestation. These conditions share a common clinical and psychosocial trajectory: a desired pregnancy that ends unexpectedly in the second trimester, necessitating surgical or medical evacuation and precipitating considerable grief reactions and a challenging psychological adaptation process.

For affected women, this experience constitutes a highly stressful life event that causes profound psychological shock, disrupts family relationships, and triggers persistent grief responses (Lafarge et al., 2014). Grief is a holistic reaction to significant loss, involving physiological, psychological, and behavioral dimensions (Worden, 2018). Women show significant heterogeneity in grief responses, shaped by coping strategies. Some use maladaptive coping mechanisms such as persistently immersing themselves in the pain of pregnancy loss, which may lead to prolonged grief disorder (Bryant et al., 2023). In contrast, others transform initial grief into post-traumatic growth (PTG) through adaptive cognitive reframing (Lafarge et al., 2017). Through this transformation, women can alleviate emotional distress and rebuild confidence (Tedeschi and Calhoun, 2004). Therefore, healthcare providers should offer personalized interventions that guide women from traumatic grief toward adaptive growth.

Current psychological interventions for women undergoing TOP are predominantly symptom-focused. Cognitive behavioral therapy (CBT), for instance, has demonstrated clinical efficacy in alleviating traumatic grief responses in this population (Kersting et al., 2011). However, such treatment-oriented approaches, which are designed for established disorders and typically delivered by mental health specialists, may be less suitable for the substantial subset of TOP women who experience significant psychological distress but do not yet meet diagnostic criteria. Their primary need is preventive support, not clinical treatment. Moreover, most interventions are delivered only during hospital admission, leaving the post-discharge puerperium, a critical window when weakened social support can facilitate the transition from normal to pathological grief, largely unaddressed (Korenromp et al., 2009). Consequently, a preventive, continuous-care intervention specifically designed for women who experience grief within the typical range for perinatal loss (Lasker and Toedter, 2000), yet remain at risk of developing prolonged grief disorder without early support (Kersting et al., 2007), would address a significant gap in current care.

Preventive intervention, by definition, aims to intervene proactively before distress escalates into disorder. Positive psychology aligns precisely with this goal (Kobau et al., 2011). Seligman's PERMA model (Seligman, 2011) provides a systematic framework for such proactive intervention. Based on this model, we developed a WeChat-based positive psychological intervention (PPI), a structured, skill-building program that systematically cultivates positive emotions, engagement, and meaning, to provide preventive and continuous care for women during the puerperium after TOP.

The primary aim of this randomized controlled trial was to evaluate the efficacy of this intervention in improving psychological adaptation among TOP women during the puerperium. We hypothesized that, compared to routine care alone, the intervention would significantly reduce grief, promote post-traumatic growth, and enhance subjective wellbeing, with effects sustained over a 1-month and 3-month follow-up period. This digital program bridges the critical gap between inpatient obstetric care and specialized mental health services, offering a scalable, low-resource model that could inform future bereavement care during the puerperium.

## 2 Participants and method

### 2.1 Study design

The study employed a randomized controlled trial design with longitudinal repeated measures to assess the efficacy of a 4-week PERMA model-based PPI program for women undergoing TOP. The study protocol and reporting strictly adhered to the Consolidated Standards of Reporting Trials (CONSORT) statement (Schulz et al., 2010).

The study employed computer-generated simple randomization, which was performed by an independent data manager who was not involved in data collection, intervention implementation, or outcome assessment. The randomization was executed using IBM SPSS Statistics software (version 27.0) following these specific procedures:

1. A unique Enrollment ID (1–118) was assigned to each of the 118 enrolled participants according to their order of admission;

2. The RV.UNIFORM(0,1) function in SPSS was used to generate a random number between 0 and 1 for each ID;

3. All generated random numbers were sorted in ascending order to create the final randomization sequence;

4. The first 59 Enrollment IDs in the sequence were allocated to the intervention group, and the remaining 59 were allocated to the control group.

To strictly implement allocation concealment, this study employed the Sequentially Numbered Opaque Sealed Envelopes method. The independent data manager prepared 118 opaque envelopes, with only a unique enrollment ID (1–118) corresponding to the participant's order of admission indicated on the outside of each envelope. The corresponding allocation information was concealed inside. The sealed envelopes were stored collectively in a locked cabinet, with the key kept solely by the data manager. After a participant completed the enrollment procedure, the researcher unsealed the corresponding envelope in sequential order to reveal the group assignment.

To minimize performance and measurement bias, the study adopted a partial blinding design with the following measures:

1. Assessor Blinding: All outcome assessments were conducted by an independent data evaluator who was blinded to group assignments and did not participate in any intervention procedures.

2. Intervention Implementer Blinding: Participant information was separately distributed by the data manager to the research teams of the intervention and control group. Personnel in each group were unaware of allocation details for participants in the other group.

It should be noted that although participants were not explicitly informed of their group assignment, complete blinding of participants was not fully achievable due to the personalized and interactive nature of the PERMA model-based positive psychological intervention. Additionally, spatial segregation between the intervention area and the control group wards was implemented to further reduce potential biases.

### 2.2 Participants and setting

Participants in this study were recruited from women who underwent TOP at a tertiary Grade A hospital. All research procedures were strictly initiated after ethical approval was obtained (Approval No. 2023KY123). The formal enrollment period for this study was from August 31, 2023, to March 20, 2024. The sample size was calculated based on the two-sample mean comparison formula (Yan et al., 2022), with the perinatal grief scale score as the primary outcome measure:


$$\begin{array}{l} n_{1}=n_{2}=2\times {\left[\frac{\left(\mu_{\alpha }+\mu_{\beta }\right)}{\delta /\sigma }\right]}^{2}+\frac{1}{4}{\mu_{\alpha }}^{2} \end{array}$$ (1)

Notes: *n*_1_, *n*_2_ = sample size required for the intervention and control group, respectively;

μ_α_ = Z-score for a two-tailed Type I error rate α (set at 1.96 for α = 0.05);

μ_β_ = Z-score for the desired statistical power (1-β) (set at 1.282 for 90% power, i.e., β = 0.10);

δ = anticipated mean difference between groups;

σ = anticipated standard deviation;

δ*/*σ = standardized effect size.

Based on a pilot study of 20 participants, the effect size (δ*/*σ) of 0.68 was determined. With a two-sided α of 0.05 and power (1-β) of 0.90, the calculated sample size was 49 participants per group. To account for a potential 20% attrition rate, the study recruited 59 participants per group (total *N* = 118).

Inclusion criteria: (1) Gestational age ≥ 12 weeks; (2) Age ≥ 20 years; (3) Confirmed diagnosis of severe fetal anomaly, intrauterine fetal death, or missed abortion; (4) Normal cognitive function and the ability to independently complete questionnaires; (5) Voluntarily provided written informed consent. Exclusion criteria: (1) History of psychiatric disorders or language/communication barriers; (2) Presence of major disease; (3) Experienced other major life stressors within the preceding 6 months.

Among 135 screened participants, 6 did not meet the inclusion criteria, 8 refused to participate, and 3 were excluded for other reasons. Ultimately, 118 eligible participants provided written informed consent and were enrolled in the study.

### 2.3 Ethical approval and consent

The study protocol was approved by the Ethics Committee of Fujian Maternity and Child Health Hospital (Approval No. 2023KY123) in accordance with the ethical principles of the Declaration of Helsinki. The trial was registered with the Chinese Clinical Trial Registry (Registration No. ChiCTR2400087627). Written informed consent was obtained from all participants. Furthermore, to fully safeguard their rights, they were explicitly informed of their unrestricted right to withdraw from the study at any stage without providing justification. All participants were assured of strict data confidentiality throughout the study.

Psychological safety and referral protocol: Given that participants were women who underwent TOP, a psychological safety monitoring protocol was established. Intervention nurses would refer participants to the study psychotherapist if participants reported severe psychological symptoms or showed scores exceeding established clinical thresholds on standardized grief measures (see Section 2.6). The protocol required prompt clinical evaluation, and if indicated, referral to a licensed psychological counselor for further assessment and support. This pathway was explained during informed consent. No participants required referral to psychological services during the study.

### 2.4 Control group

The control group received the routine postpartum care protocol: Obstetrician-gynecologists and the nursing team provided standardized care to patients, including postpartum health education (covering disease-related knowledge, medication guidance, dietary and exercise recommendations), psychological counseling and outpatient follow-up visits (scheduled at 2 weeks and 1 month postpartum), with supplemental continuous postpartum consultation and guidance delivered through the WeChat platform.

### 2.5 Intervention group

Participants in the intervention group received an additional 4-week WeChat-based PERMA model positive psychological intervention (WB-PERMA PPI) in addition to the identical routine care provided to the control group. The intervention was implemented by a multidisciplinary team consisting of specialist nurses, an OB/GYN, a genetic counselor, and a psychotherapist (a detailed description of the team composition and the responsibilities of each member can be found in Supplementary File S3). The intervention framework, initially developed through literature analysis to encompass four core themes, underwent protocol refinement through pilot testing and three rounds of expert deliberations. Specific implementation details are provided in Table 1.

**Table 1:** Implementation protocol of the PERMA model-based PPI.

Implementation phase	Core theme	Key intervention components	Format/duration
Week 1 (inpatient phase)	Cultivating positive emotions	1. Psychological assessment and emotion release 2. Guided positive cognitive reconstruction 3. Lamaze breathing exercises (10–20 times/day) 4. Positive emotion cultivation train	Face-to-face session + Video instruction (45–60 min/session)
Week 2 (home-based phase)	Enhancing flow experience	1. Flow state immersion in strengthen domains 2. Meditation training 3. *Baduanjin* exercises (traditional Chinese qigong)	WeChat Communication (30–50 min/session)
Week 3 (home-based phase)	Gratitude and building positive relationships	1. Adversity acceptance with integrated gratitude 2. Fertility anxiety counseling 3. Social support network building 4. Peer experience sharing	WeChat Communication (30–50 min/session)
Week 4 (home-based phase)	Discovering life meaning	1. Meaning analysis of traumatic events and psychological growth 2. Strength-based training to enhance self-efficacy 3. Life goal reorientation	WeChat Communication (30–50 min/session)

The 59 participants in the intervention group were divided into 6 cohorts based on admission time (5 cohorts with 10 participants each and 1 cohort of 9 participants). The protocol followed a phased progressive model with interventions implemented twice weekly.

**Phase 1 (Inpatient Phase):**

Theoretical learning and practical guidance were conducted in a dedicated instructional room within the ward. Concurrently, participants were provided with a “Happiness Journal” to facilitate daily recording of positive events.

**Phase 2–4 (Home-based Phase):**

A remote intervention system was established via the WeChat platform, delivering standardized intervention content through scheduled weekly updates. Continuity of health management was maintained through video tutorials and online Q&A sessions.

**Figure 1:**
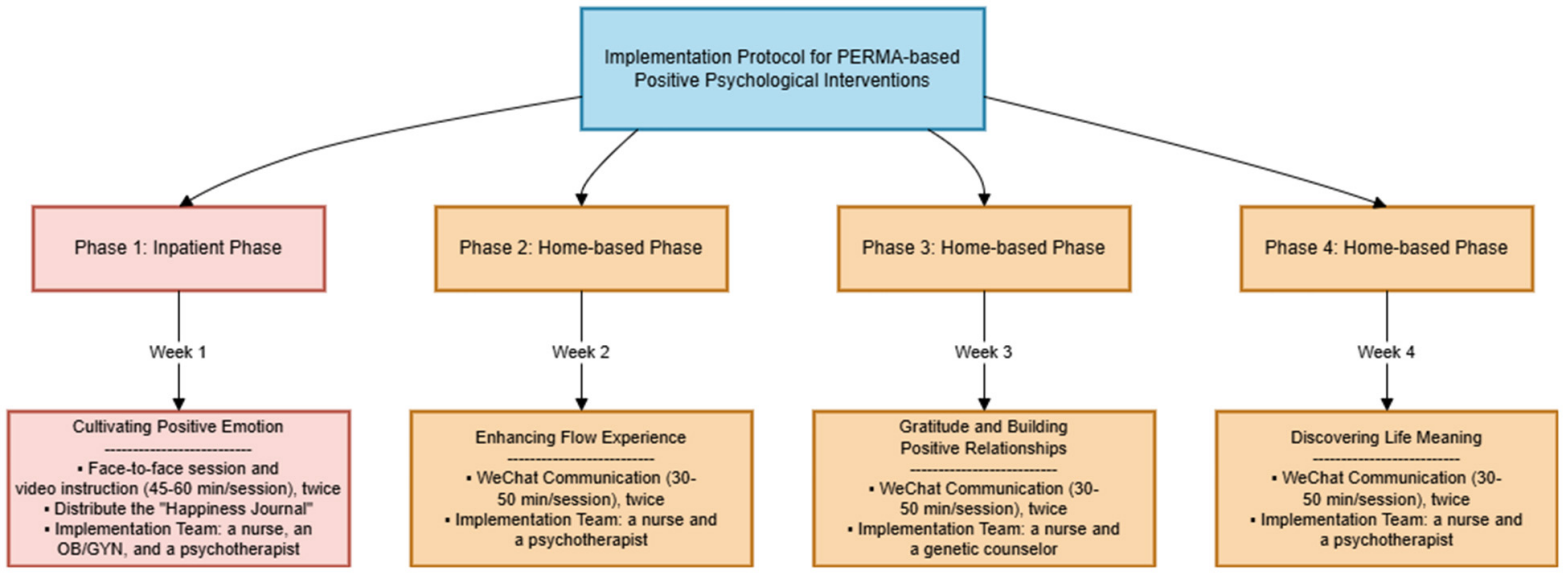
Flowchart of the PERMA model-based intervention protocol.

To ensure intervention expertise and specificity, a dynamic team rotation mechanism was implemented across phases. Each phase was independently managed by distinct intervention teams composed of personnel who had completed standardized training. The complete flowchart is illustrated in Figure 1.

A multi-level strategy to ensure intervention fidelity was implemented to guarantee the intervention was delivered as planned and to assess participant adherence. The specific strategies were as follows:

1. Intervention Fidelity Monitoring Strategy:

During the home-based intervention phase (Weeks 2–4), participants were required to check in via a WeChat group before each session. At the end of each session, a sharing segment was arranged to invite participants to exchange insights or discuss content from their “Happiness Journal,” which served as evidence of their interactive engagement.

For participants who missed a session due to scheduling conflicts (e.g., childcare responsibilities), all course materials remained accessible in the WeChat group for later review. For those who missed a session for other reasons, the intervention team followed up individually via private message to identify barriers and provide support.

2. Implementation Deviations and Countermeasures:

Some participants deviated from the protocol during the intervention. The primary challenges included difficulty attending sessions on time due to scheduling conflicts (e.g., picking up children, assisting with homework). Additionally, adherence to certain home-based activities (e.g., mindfulness meditation, *Baduanjin* exercise) declined when completed independently. To minimize the impact of these deviations on intervention outcomes, the following measures were implemented:

(1) All course materials were retained in the WeChat group, allowing participants to review content at their convenience;

(2) Family members were actively encouraged to join the group to enhance social support through collective family participation, thereby boosting participant motivation;

(3) A full-attendance reward mechanism was established, offering participants who completed the entire intervention free follow-up counseling sessions to encourage long-term engagement.

3. Provider Fidelity

All intervention team members completed standardized training before the study began. Weekly team debriefings were conducted to review protocol adherence and address any implementation challenges.

4. Participant Adherence Data

Of the 59 participants in the intervention group, 52 (88.1%) completed all 8 intervention sessions (2 in-person + 6 online), representing an overall attrition rate of 11.9% (7/59).

The intervention was delivered in eight standardized sessions (two per week) targeting the five PERMA domains via a phased format combining face-to-face (inpatient) and WeChat-based (home-based) delivery, using structured materials including a Happiness Journal and other educational media. It was supported by a three-module provider training program and a multi-level fidelity monitoring system. Detailed session content, materials, training curricula, and fidelity evaluation procedures are provided in Supplementary File S5.

### 2.6 Measures

The study employed four scales for data collection, which were completed by participants.

1. **General Information Questionnaire:** This self-developed questionnaire comprises two sections:

Section 1: Demographic characteristics—including age, education level, occupation type, personality traits, and family relationships.

Section 2: Obstetric information—including pregnancy intentions, conception methods, history of adverse pregnancies, gestational age, and obstetric history.

2. **Perinatal Grief Scale (PGS):** Originally developed by Potvin et al., 1989 and adapted into Chinese by Xu (2014), this scale assesses grief responses in populations who have experienced fetal loss. The Chinese version scale contains 33 items across three dimensions: Current Grief (11 items), Difficulty Coping (11 items), and Despair (11 items). It uses a 5-point Likert scale (1 = “strongly agree” to 5 = “strongly disagree”). Total scores range from 33 to 165, with higher scores indicating more severe grief. A total score of 91 or above suggests potential prolonged grief disorder. In this study, the reliability test of the sample data showed that the Cronbach's α coefficient of the scale was 0.875, indicating good reliability of the scale.

3. **Chinese Post-traumatic Growth Inventory (C-PTGI):** Adapted into Simplified Chinese by Wang et al., 2011, this scale assesses PTG levels. It comprises 20 items across five dimensions: Relating to Others, New Possibilities, Personal Strength, Spiritual Change, and Appreciation of Life. Each item is rated on a 6-point Likert scale (0 = “No Change” to 5 = “Significant Change”). Total scores range from 0 to 100, with higher scores reflecting a greater level of PTG. In this study, the measured Cronbach's α coefficient for the scale was 0.869, indicating that the measurement results of the scale were reliable.

4. **Index of Wellbeing (IWB):** The scale was originally developed by Campbell (1976) and later adapted into a Chinese version by Wang et al., 1999 to assess an individual's current SWB. It comprises two components: Index of General Affect (8 items) and Index of Life Satisfaction (1 item). Items are scored on a 7-point Likert scale. The total score ranges from 2.1–14.7 and is categorized into three levels: low wellbeing (2.1–6), moderate wellbeing (6.1–10), and high wellbeing (10.1–14.7). The analysis of the collected data in this study revealed a Cronbach's α coefficient of 0.861 for the overall scale, suggesting that the scale maintained good reliability within the context of this study.

### 2.7 Data collection

During the baseline assessment phase, uniformly trained data collectors guided participants to complete scale evaluations using standardized procedures after enrollment. Data collection was conducted through two methods: the General Information Questionnaire was filled out in paper form, while the PGS, C-PTGI, and IWB were administered via electronic questionnaires created on the professional online survey platform “Wenjuanxing” (WJX, www.wjx.cn). At the 1-month and 3-month follow-up time points after completion of the 4-week intervention, nurses assigned to the intervention group and those in the control group distributed standardized electronic questionnaire links to participants via WeChat separately. Participants accessed and completed the assessments online through encrypted links. All data underwent double independent data entry and cross-verification to ensure accuracy.

### 2.8 Statistical methods

Data analysis was performed using IBM SPSS software (version 27.0) with the following procedures:

1. Baseline demographic and clinical characteristics were summarized using descriptive statistics. Continuous variables were presented as mean ± standard deviation (M ± SD), while categorical variables were described using frequency (percentage).

2. The scores of all outcome measures (grief, PTG, and SWB) at T0, T1, and T2 were presented descriptively as M ± SD along with the sample size (*n*).

3. The intention-to-treat (ITT) principle was implemented in primary outcome analysis to preserve the original randomized group allocation to control potential selection bias (Gravel et al., 2007). Longitudinal intervention effects on TOP women's mental health indicators (including grief, PTG, and SWB) were evaluated using generalized estimating equations (GEE) for repeated measures analysis (Ma et al., 2012).

GEE analysis was performed using robust standard errors, with the following specifications:

**Outcome variables**: Scores of PGS, C-PTGI, and IWB, respectively.

**Predictor variables**: The primary predictors were group (intervention vs. control), time (T0, T1, T2), and their interaction (group × time).

**Distribution and link function**: As the dependent variables are continuous, they were specified to follow a normal distribution with an identity link function.

**Working correlation matrix**: Based on comparison of Quasi-likelihood under the Independence model Criterion values, the exchangeable correlation structure was identified as optimal for handling the within-subject correlations arising from repeated measurements.

These model specifications were chosen to appropriately address the longitudinal nature of the data and to produce robust estimates.

**Handling of TOP indications**: Indications for TOP (fetal anomaly, intrauterine fetal death, missed abortion) were recorded as baseline clinical characteristics. Given that randomization was expected to balance prognostic factors across groups and that the number of intrauterine fetal death cases was very small (*n* = 5), indication type was not entered as a covariate in the primary GEE models. No subgroup analyses by indication were planned, as the study was not powered for such comparisons.

4. To examine the robustness of the study findings, a per-protocol (PP) analysis was also performed. The PP analysis included only those participants who had completed all intervention sessions and provided valid data at each follow-up assessment. PP analysis was intended to corroborate the ITT analysis and thereby further enhance the reliability of the study conclusions.

## 3 Results

**Figure 2:**
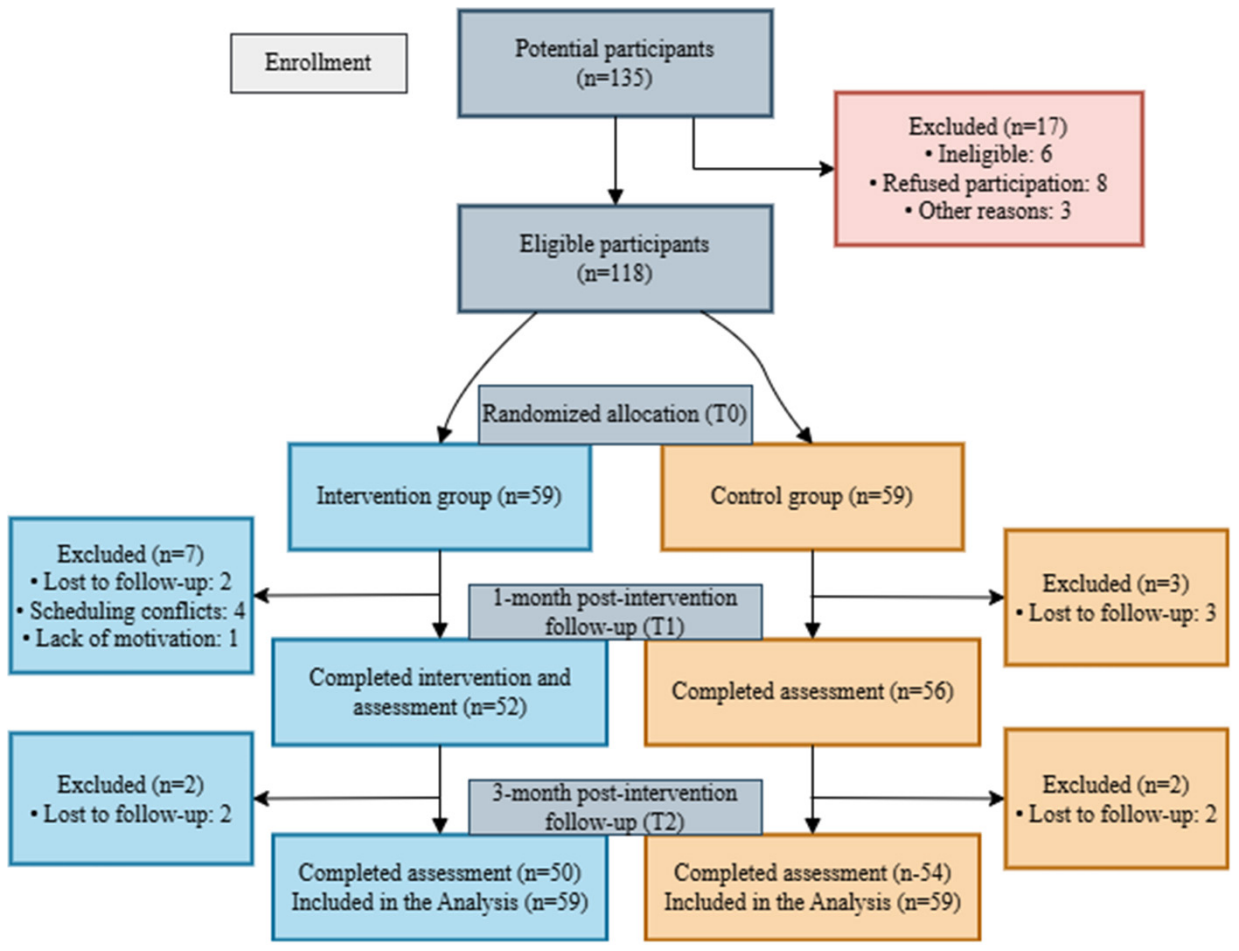
CONSORT flow diagram.

The study enrolled 118 puerperal women who had previously undergone TOP as study participants, who were randomly allocated into an intervention group and a control group (59 participants each). During the baseline assessment phase, all enrolled participants completed basic data collection. The study flowchart and follow-up details are shown in Figure 2 (CONSORT flow diagram).

### 3.1 Baseline characteristics by group

The baseline demographic and clinical characteristics of the intervention and control groups are presented in Table 2. As expected under randomization, the two groups showed no significant differences in any baseline characteristics. The distribution of TOP indications was well balanced between groups (χ^2^ = 0.29, *p* = 0.86).

**Table 2:** Baseline demographic and clinical characteristics of participants by group.

Variable	Category	Intervention group (*n* = 59) M ±SD/*n* (%)	Control group (*n* = 59) M ±SD/*n* (%)
Age		31.49 ± 4.36	31.12 ± 4.32
Education level	Junior high school	5 (8.5)	7 (11.9)
	High school/technical school	7 (11.9)	9 (15.3)
	College/bachelor's degree	39 (66.1)	32 (54.2)
	Postgraduate	8 (13.5)	11 (18.6)
Occupation	Staff	24 (40.8)	19 (32.2)
	Farming	11 (18.6)	18 (30.5)
	Self-employed	10 (16.9)	8 (13.6)
	Others	14 (23.7)	14 (23.7)
Personality	Introverted	8 (13.6)	11 (18.6)
	Moderate	41 (69.5)	37 (62.8)
	Extroverted	10 (16.9)	11 (18.6)
Family relationship	Good	46 (78.0)	45 (76.3)
	Neutral	9 (15.3)	10 (16.9)
	Poor	4 (6.8)	4 (6.8)
Pregnancy intention	Planned	39 (66.1)	40 (67.8)
	Unplanned	20 (33.9)	19 (32.2)
Conception method	Natural conception	47 (79.7)	45 (76.3)
	Assisted reproduction	12 (20.3)	14 (23.7)
Indications for TOP	Fetal anomaly	31 (52.5)	30 (50.8)
	Intrauterine fetal death	3 (5.1)	2 (3.4)
	Missed abortion	25 (42.4)	27 (45.8)
Adverse reproductive history	None	23 (39.0)	24 (40.7)
	Once	26 (44.1)	24 (40.7)
	≥ 2 times	10 (16.9)	11 (18.6)
Gestational age		13.83 ± 1.46	13.51 ± 1.41
Obstetric history	Yes	24 (40.7)	27 (45.8)
	No	35 (59.3)	32 (54.2)

### 3.2 Missing data and analysis

During the follow-up period of this study, partial data were missing. At 1-month after intervention (T1), 2 participants were lost to follow-up in the intervention group, with an additional 5 participants failing to complete all intervention measures (due to scheduling conflicts or other reasons) while still completing scale assessments. In the control group, 3 participants were lost to follow-up at T1. By the 3-month follow-up time point (T2), 2 additional participants were lost in the intervention group and 2 in the control group. The overall missing data rate was 10.2% (12/118), comprising 5.9% (7/118) lost to follow-up and 4.2% (5/118) who partially participated but completed assessments.

We further analyzed the pattern of missing data by comparing baseline characteristics between completers and non-completers. No statistically significant differences were observed in any baseline variables between the two groups (all *p* > 0.05), suggesting that the missing data were likely missing at random. Therefore, an ITT analysis using GEE was employed in this study, which included all randomized participants in the analysis to minimize bias caused by missing data.

**Figure 3:**
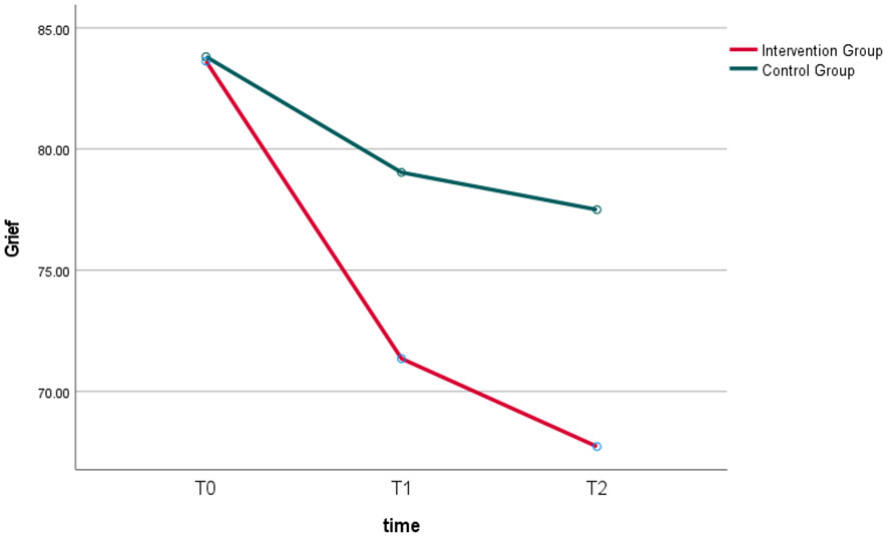
Intervention vs. control: grief (PGS) across T0–T2.

**Figure 4:**
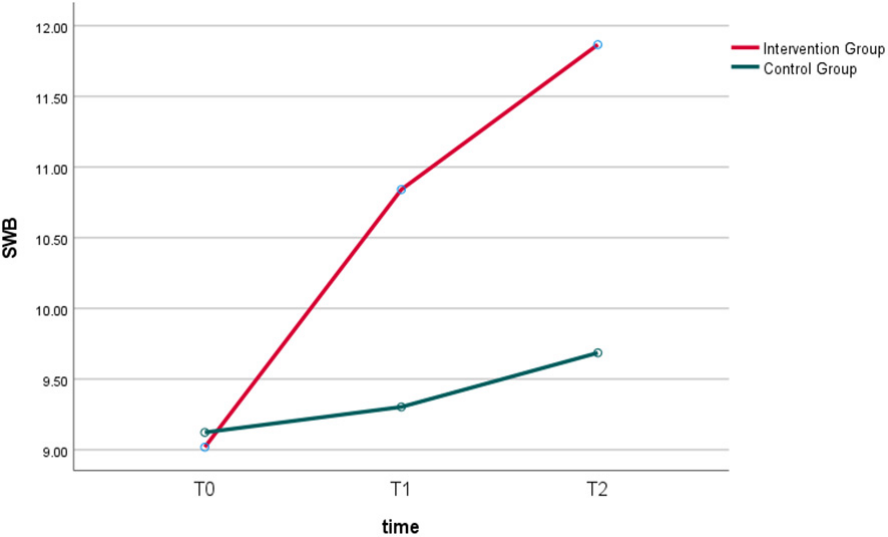
Intervention vs. control: PTG (C-PTGI) across T0–T2.

**Figure 5:**
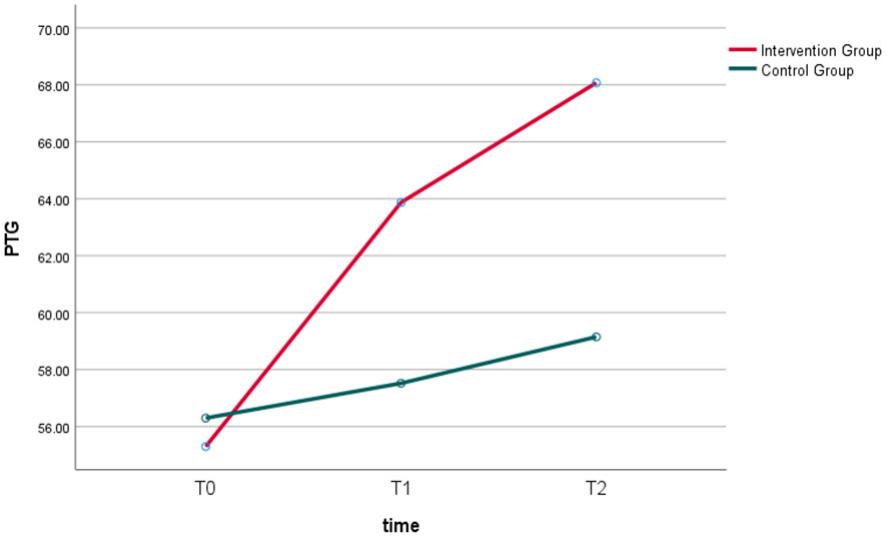
Intervention vs. control: SWB (IWB) across T0–T2.

Despite partial data absence, the samples that completed the follow-up still provided valuable longitudinal data. The descriptive data for the two groups of patients on grief (PGS), post-traumatic growth (C-PTGI), and subjective wellbeing (IWB) at the three time points (T0, T1, T2) are presented in Table 3 and visually summarized in Figures 3–5.

**Table 3:** Descriptive data of outcome measures at baseline and follow-up time points.

Outcome measure	Group	T0 M ±SD (*n*)	T1 M ±SD (*n*)	T2 M ±SD (*n*)
Grief (PGS)	Intervention	83.63 ± 6.98 (59)	71.35 ± 5.18 (57)	67.72 ± 6.64 (55)
	Control	83.82 ± 7.60 (59)	79.04 ± 6.02 (56)	77.50 ± 5.73 (54)
PTG (C-PTGI)	Intervention	55.30 ± 5.46 (59)	63.87 ± 8.65 (57)	68.07 ± 7.97 (55)
	Control	56.30 ± 6.18 (59)	57.52 ± 5.26 (56)	59.15 ± 4.12 (54)
SWB (IWB)	Intervention	9.02 ± 1.60 (59)	10.84 ± 2.80 (57)	11.87 ± 1.72 (55)
	Control	9.12 ± 1.70 (59)	9.30 ± 1.75 (56)	9.69 ± 1.12 (54)

Data are presented as mean ± standard deviation (SD). *n* indicates the number of participants completing the assessment at each time point.

### 3.3 Effect of WeChat-based PERMA model PPI on grief, PTG, and SWB

The results of the WB-PERMA PPI on grief, PTG, and SWB are summarized in Table 4. GEE were employed to analyze the interaction effects between the intervention and time variables, using the control group as the reference.

**Table 4:** Effect of PERMA model-based PPI on grief, PTG, and SWB using the GEE model.

Variable	Regression coefficient β	Standard error	95% CI		*p*-value	Cohen's *d*
			Lower	Upper		
PGS						
Intervention vs. control at T0	−1.31	1.46	−4.17	1.56	0.371	—
T1 vs. T0	−5.08	0.51	−6.07	−4.08	< 0.001^***^	—
T2 vs. T0	−6.81	0.83	−8.44	−5.18	< 0.001^***^	—
Intervention × T1	−7.03	1.20	−9.37	−4.68	< 0.001^***^	−1.37
Intervention × T2	−8.47	1.31	−11.03	−5.91	< 0.001^***^	−1.58
C-PTGI						
Intervention vs. control at T0	−2.03	1.19	−4.23	0.16	0.069	—
T1 vs. T0	0.94	0.29	0.37	1.51	0.001^**^	—
T2 vs. T0	2.39	0.43	1.54	3.23	< 0.001^***^	—
Intervention × T1	7.98	1.40	5.24	10.72	< 0.001^***^	0.89
Intervention × T2	10.96	1.49	8.03	13.89	< 0.001^***^	1.40
IWB						
Intervention vs. control at T0	−0.06	0.31	−0.66	0.54	0.845	—
T1 vs. T0	0.17	0.21	−0.24	0.57	0.419	—
T2 vs. T0	0.66	0.20	0.28	1.05	0.001^**^	—
Intervention × T1	1.59	0.30	1.00	2.17	< 0.001^***^	0.66
Intervention × T2	2.24	0.29	1.67	2.81	< 0.001^***^	1.50

^**^p < 0.01, ^***^p < 0.001; CI, Confidence Interval. T0: Baseline; T1: 1-month post-intervention follow-up. T2: 3-month post-intervention follow-up. Intervention × T1: Interaction effect of the intervention group at 1-month follow-up. Intervention × T2: Interaction effect of the intervention group at 3-month follow-up. PGS, Perinatal Grief Scale; C-PTGI, Chinese Post-traumatic Growth Inventory; IWB, Index of Wellbeing.

The study results demonstrated the following findings across different psychological dimensions:

#### 3.3.1 Grief (PGS)

At baseline (T0), the intervention group scored 1.31 points lower than the control group, but this difference was not statistically significant (*p* = 0.371). Over time, the intervention group exhibited a significant reduction in grief levels. The interaction effects for the intervention group were −7.03 points at the 1-month follow-up (T1) (*p* < 0.001) and −8.47 points at the 3-month follow-up (T2) (*p* < 0.001). When combined with the time main effects, the total grief scores in the intervention group decreased by 12.11 (sum of −5.08 and −7.03) points at T1 and 15.28 (sum of −6.81 and −8.47) points at T2 compared to baseline, indicating significantly superior outcomes relative to the control group. The Cohen's *d* values for the between-group difference were −1.37 at T1 and −1.58 at T2.

#### 3.3.2 PTG (C-PTGI)

No significant baseline differences were observed between the groups (Δ = −2.03, *p* = 0.069). Nevertheless, significant intervention-by-time interaction effects emerged: the PTG scores of the intervention group were higher than those of the control group by 7.98 points at T1 (*p* < 0.001) and 10.96 points at T2 (*p* < 0.001). When combined with the time main effects, the intervention group showed cumulative increases in PTG scores of 8.92 (sum of 0.94 and 7.98) points at T1 and 13.35 (sum of 2.39 and 10.96) points at T2 compared to baseline, reflecting a sustained progressive improvement in PTG. Cohen's *d* values were 0.89 at T1 and 1.40 at T2.

#### 3.3.3 Subjective wellbeing (IWB)

At baseline (T0), no significant differences in SWB scores were observed between the two groups (Δ = −0.06, *p* = 0.845). The control group displayed no significant change at T1 (*p* = 0.419), but showed a 0.66-point improvement at T2 (*p* < 0.01). In contrast, the intervention group demonstrated strong interaction-by-time interaction effects: β = 1.59 at T1 (*p* < 0.001) and β = 2.24 at T2 (*p* < 0.001). When considering the time main effect at T2 (*p* < 0.001), the intervention group's total SWB score increased by 2.90 (sum of 0.66 and 2.24) points from baseline at T2, significantly outperforming the control group. Cohen's *d* values were 0.66 at T1 and 1.50 at T2.

### 3.4 Robustness analysis

The results of the PP analysis (see Supplementary Material S4) were consistent with the ITT analysis. At both T1 and T2 time points, the between-group differences in grief (PGS), PTG (C-PTGI), and SWB (IWB) remained statistically significant (all *p* < 0.01). Moreover, the effect sizes highly aligned with those observed in the ITT analysis. This consistency reinforces the reliability and robustness of the intervention effects demonstrated in this study.

## 4 Discussion

The study results demonstrated that the WB-PERMA PPI significantly reduced grief levels in TOP women. The intervention group showed significantly lower grief levels than the control group at both follow-ups, with large effect sizes. These sustained effects may stem from the two-phase intervention design: establishing a foundation for positive emotions through face-to-face guidance during hospitalization, followed by continuous cognitive restructuring and behavioral reinforcement via the WeChat platform post-discharge. Additionally, the “Happiness Journal” component effectively alleviated excessive self-blame regarding pregnancy termination by encouraging daily recording of positive experiences, thereby enhancing adaptive coping with traumatic events. Compared with traditional CBT, this intervention model showed superior long-term maintenance effects. In Kersting's randomized controlled trial with bereaved mothers (Kersting et al., 2011), while CBT significantly reduced grief levels immediately post-treatment (ICG scores decreased from 39.5 to 30.1, *p* < 0.001), scores showed slight rebound at 3-month follow-up (30.1 → 30.5), though this fluctuation might reflect random error (*p* = 0.811). In contrast, our study observed continuously increasing effect sizes at T2, suggesting the PERMA model's potential advantages in sustaining long-term efficacy. This difference may originate from distinct therapeutic pathways: traditional CBT focuses on modifying negative cognitions through cognitive restructuring, whereas PPIs emphasize PTG and SWB enhancement. According to Fredrickson's broaden-and-build theory of positive emotions (Fredrickson, 2001), cultivating positive emotions expands cognitive resources and psychological resilience, fostering sustained adaptive behaviors. This self-reinforcing psychological mechanism helps establish a virtuous cycle of mental health, potentially better addressing the post-trauma recovery needs of women experiencing TOP.

The study further revealed that the intervention group demonstrated a sustained increase in PTG over time, with effect sizes increased over time from moderate to large. This finding aligns with Qian's study conclusions regarding expressive writing (EW) interventions promoting PTG (Qian et al., 2021). From the perspective of cognitive processing mechanisms, the intervention effects may be attributed to the systematic guidance of cognitive restructuring in the intervention program: Firstly, the “Cultivating Positive Emotions” module effectively facilitated the transformation of rumination from emotional immersion to rational analysis through negative emotion regulation. Secondly, the “Discovering Life Meaning” curriculum guided participants to shift cognitive focus from short-term bereavement trauma to long-term personal development goals through goal-oriented restructuring, thus progressively forming deliberate rumination.Additionally, the “Gratitude and Building Positive Relationships” training reinforced positive feedback of the family support system, further suppressing intrusive rumination, while enhancing deliberate rumination (Ye et al., 2024). Notably, as deliberate rumination has been identified as a crucial predictor and mediator of PTG (Lafarge et al., 2020), its stepwise enhancement throughout this intervention may constitute the mediating pathway for the continuous improvement in PTG levels.

Furthermore, this study observed a progressive enhancement of PTG effects over time (T2 > T1), demonstrating a significant divergence from the effect attenuation observed at 1-month follow-up in Qian's study (Qian et al., 2021). Specifically, Qian's study revealed that the intervention group's PTG scores were significantly higher than the control group by 8.68 points immediately post-intervention (*p* = 0.003), but this difference decreased to 6.78 points (*p* = 0.014) at 1-month follow-up, indicating diminished effect intensity. This discrepancy may stem from differences in intervention models: This study's intervention implemented through the WeChat platform featured continuity (twice-weekly resource delivery over 4 weeks) and interactivity (online group discussions), whereas Qian's EW intervention comprised three intensive writing sessions (total 45 min) during hospitalization. According to the PTG model (Tedeschi and Calhoun, 2004), PTG achievement requires a sequential progression through “emotional catharsis → cognitive integration → meaning reconstruction”. While short-term interventions may generate immediate effects through emotional catharsis, the absence of sustained cognitive reinforcement mechanisms might lead to effect attenuation over time. In contrast, this study employed phased positive resource inputs that not only facilitated immediate emotional regulation but also promoted gradual cognitive restructuring of traumatic events through phased cognitive training and social reinforcement mechanisms, thereby achieving cumulative consolidation of PTG effects.

The findings of this study revealed that the WB-PERMA PPI significantly enhanced SWB. Longitudinal data analysis indicated that the intervention group's IWB scores increased significantly at T2 compared to T0 (*p* < 0.01), with the intervention effect showing a clear increasing trend from 1 month to 3 months. This trend aligns with the conclusion from Corno's study that “PPIs can improve wellbeing through multiple pathways” (Corno et al., 2019). The PERMA-based intervention in this study systematically enhanced SWB through multidimensional strategies: Firstly, the “Happiness Journal” guided participants to actively document daily positive events, heightening awareness of pleasurable and grateful emotions. Secondly, practices such as meditation and *Baduanjin* promoted immersive experiences while reducing negative emotional interference. Meanwhile, gratitude training and trauma-focused discussions facilitated cognitive restructuring, and WeChat-based social groups established supportive interpersonal networks. Additionally, participants explored the meaning of pregnancy termination experiences and set life goals to reorient life direction. Repeated measures and control group analysis confirmed that these strategies effectively improved psychological comfort, perceived social support, and self-efficacy, ultimately promoting the holistic enhancement of SWB.

Beyond the specific changes in grief, PTG, and SWB, the clinical significance of the WB-PERMA PPI warrants explicit discussion. The clinical significance of this study does not lie in replacing professional psychotherapy, but in bridging the transitional gap between obstetric nursing and specialized psychological intervention. Most women who undergo TOP experience a subclinical distress state after discharge—they neither meet diagnostic criteria for mental disorders (thus requiring no immediate referral to psychiatric services), nor are their needs for psychological resilience reconstruction adequately addressed by routine postnatal follow-up.

Lasker and Toedter (2000) reported that 95% of women with perinatal loss scored between 78 and 91 on the PGS total score, with a score above 91 indicating high-level grief. In this study, the baseline PGS score of the intervention group was 83.63, falling within that range, indicating significant but non-extreme grief reactions. However, without intervention, longitudinal studies by Kersting et al. (2007) and Dawood et al. (2024) have shown that approximately 25% of such women develop pathological grief or mental disorders within 4 to 14 months, suggesting that natural recovery is not universal.

Following the 4-week PERMA-based positive psychological intervention, the PGS total score in the intervention group decreased from a baseline of 83.63 to 71.35 at 1 month and 67.72 at 3 months, falling well below the threshold of the high-level grief (91). These changes indicate sustained improvement in self-reported grief severity. As this study examined psychological adaptation during the early puerperium rather than treatment of diagnosed disorders, these score changes reflect preventive support efficacy within the measured domain, offering a low-resource care option for women following pregnancy termination.

Therefore, the nurse-led PERMA-based positive psychological intervention is conceptualized as a preventive support program that targets the critical puerperium period. This period represents a window of heightened vulnerability to prolonged grief, as social support typically weakens during the transition from hospital to home, making early intervention essential for facilitating adaptive psychological adjustment and promoting post-traumatic growth. This is a health promotion model rather than a pathology-correction model, providing a scalable, low-cost transitional support option for TOP women. Moreover, the intervention also significantly improved patients' PTG and SWB, suggesting that it may yield broader psychological benefits through mechanisms such as enhancing positive emotions, sense of meaning, and interpersonal relationships.

## 5 Limitations

This study has the following limitations that warrant cautious consideration.

Firstly, this study employed self-report scales to assess grief, post-traumatic growth, and wellbeing. While the PGS provides a validated indicator of grief severity, it does not constitute a diagnostic instrument for prolonged grief disorder or other clinical conditions. Consequently, the observed score reductions reflect psychometric improvement within the present sample rather than confirmed clinical outcomes.

Secondly, the sample is characterized by geographic and institutional homogeneity, as all participants were recruited from a single tertiary Grade A hospital in an economically developed city of Eastern China. The homogeneity is particularly pronounced in aspects such as socioeconomic status, healthcare accessibility, and mental health literacy. Therefore, caution should be exercised when generalizing the findings of this study to populations in other regions, including other cities (e.g., those in Western China) as well as rural or economically underdeveloped areas, because psychological characteristics, coping strategies, and help-seeking behaviors in these regions may differ from those of the present sample, which may in turn affect the generalizability of the intervention effects. It should be emphasized that this does not mean the intervention model is inapplicable to other regions; rather, it indicates that the current empirical evidence is limited to a single-city sample, and its robustness across different populations requires further validation. Future research should include samples from multiple geographic regions (covering both cities in different areas and rural regions) and from multi-level healthcare institutions to examine the applicability and consistency of the intervention model in broader populations.

Thirdly, this intervention program deeply integrates elements of traditional Chinese culture and the digital ecosystem. In terms of cultural elements, the “Baduanjin” exercise adopted in the program is a set of Qigong movements unique to China, which lacks a direct equivalent in other cultural contexts. Therefore, if this intervention program is to be extended to other cultural settings, cultural adaptation of specific components is necessary, such as replacing them with locally acceptable mind-body practices that serve a similar function (e.g., mindful walking, yoga, or localized relaxation exercises). Regarding the digital ecosystem, this intervention program is implemented via the WeChat platform. As a super app unique to China, WeChat integrates functions such as instant messaging, group interaction, multimedia content delivery, and long-term file storage into a single platform, thereby supporting structured remote group interventions twice a week for 30–50 min per session and achieving a high level of adherence. However, this specific combination of WeChat's functions, particularly its seamless support for synchronous group chat and asynchronous access to review materials, may not be equally integrated into mainstream instant messaging platforms in other cultural contexts. Consequently, when evaluating the cross-cultural generalizability of this intervention model, its dependence on the Chinese digital ecosystem should be carefully considered.

Fourthly, a cumulative growth trend in intervention effects was observed in this study (effect sizes at T2 > T1), suggesting that the positive psychological intervention may have delayed effects or a sustained constructive process. However, the follow-up in this study lasted only up to 3 months post-intervention, and due to the lack of follow-up data at 6 months or beyond, we cannot determine whether this upward trajectory will continue to rise, stabilize, or eventually decline or rebound at later time points. Therefore, the current evidence supports only short-term efficacy, and its long-term prognostic validity remains to be verified. Future research should extend the follow-up period (e.g., to 6 or 12 months) to verify the durability of the intervention effects, with particular attention to TOP-specific time points such as the anticipated delivery date, anniversary reactions, and the decision-making period for subsequent pregnancy.

## 6 Conclusion

The study focused on puerperal psychological crises induced by TOP, developing a nurse-led PPI program based on the PERMA model. By integrating the digital support features of the WeChat platform, this intervention system demonstrated significant effects on psychological adaptation: effectively reducing traumatic grief response, promoting PTG, and enhancing SWB in women after TOP. These findings support the applicability of the WB-PERMA PPI in puerperium psychological crisis management. The sustained and increasing effects observed at 3-month follow-up suggest that a 4-week intervention program with continued digital support may produce lasting psychological benefits that persist through and beyond the puerperium. The intervention draws on positive psychology principles underlying the PERMA framework, supporting women who underwent TOP in their psychological adaptation during the transition from hospital to home. This provides an implementable pathway for establishing psychological rehabilitation systems for bereaved mothers during the puerperium.

## Data Availability

The original contributions presented in the study are included in the article/Supplementary material, further inquiries can be directed to the corresponding author/s.

## Supplementary material

Supplementary File S1 Intervention data.

Supplementary File S2 CONSORT checklist.

Supplementary File S3 Research team roles and responsibilities.

Supplementary File S4 Per-protocol analysis of outcome measures.

Supplementary File S5 Detailed intervention protocol.
