# Supplementary material for: Effects of a WeChat-based PERMA model positive psychological intervention on Chinese women after termination of pregnancy: a randomized controlled trial
Source: Front Psychol. 2026 Jul 20;17:1823918. doi: 10.3389/fpsyg.2026.1823918 (PMC13429383; doi:10.3389/fpsyg.2026.1823918)
Supplement: Supplementary File S2 — CONSORT checklist. [file Table_2.docx]

**CONSORT 2010 checklist**

| **Section/Topic** | **Item No** | **Checklist item** | **Location**  **(Section)** |
| --- | --- | --- | --- |
| **Title and**  **abstract** | 1a | Identification as a randomised trial in the title | Title |
|  | 1b | Structured summary of trial design, methods, results, and conclusions (for specific guidance see CONSORT for abstracts) | Abstract |
| **Introduction** | | |  |
| **Background and objectives** | 2a | Scientific background and explanation of rationale | Introduction |
|  | 2b | Specific objectives or hypotheses | Introduction |
| **Methods** | | |  |
| **Trial design** | 3a | Description of trial design (such as parallel, factorial) including allocation ratio | Participants and Method |
|  | 3b | Important changes to methods after trial commencement (such as 4b6.), with reasons | N/A |
| **Participants** | 4a | Eligibility criteria for participants | Participants and Method |
|  | 4b | Settings and locations where the data were collected | Participants and Method |
| **Interventions** | 5 | The interventions for each group with sufficient details to allow replication, including how and when they were actually administered | Participants and Method |
| **Outcomes** | 6a | Completely defined pre-specified primary and secondary outcome measures, including how and when they were assessed | Participants and Method |
|  | 6b | Any changes to trial outcomes after the trial commenced, with reasons | N/A |
| **Sample size** | 7a | How sample size was determined | Participants and Method |
|  | 7b | When applicable, explanation of any interim analyses and stopping guidelines | N/A |
| **Randomisation** | | |  |
| **Sequence**  **generation** | 8a | Method used to generate the random allocation sequence | Participants and Method |
|  | 8b | Type of randomisation; details of any restriction (such as blocking and block size) | Participants and Method |
| **Allocation concealment mechanism** | 9 | Mechanism used to implement the random allocation sequence (such as sequentially numbered containers), describing any steps taken to conceal the sequence until interventions were assigned | Participants and Method |
| **Implementation** | 10 | Who generated the random allocation sequence, who enrolled participants, and who assigned participants to interventions | Participants and Method |
| **Blinding** | 11a | If done, who was blinded after assignment to interventions (for example, participants, care providers, those assessing outcomes) and how | Participants and Method |
|  | 11b | If relevant, description of the similarity of interventions | N/A |
| **Statistical**  **methods** | 12a | Statistical methods used to compare groups for primary and secondary outcomes | Participants and Method |
|  | 12b | Methods for additional analyses, such as subgroup analyses and adjusted analyses | N/A |
| **Results** | | |  |
| **Participant flow (a diagram is strongly**  **recommended)** | 13a | For each group, the numbers of participants who were randomly assigned, received intended treatment, and were analysed for the primary outcome | Results |
|  | 13b | For each group, losses and exclusions after randomisation, together with reasons | Results |
| **Recruitment** | 14a | Dates defining the periods of recruitment and follow-up | Participants and Method/Results |
|  | 14b | Why the trial ended or was stopped | N/A |
| **Baseline data** | 15 | A table showing baseline demographic and clinical characteristics for each group | Results |
| **Numbers**  **analysed** | 16 | For each group, number of participants (denominator) included in each analysis and whether the analysis was by original assigned groups | Results |
| **Outcomes**  **and**  **estimation** | 17a | For each primary and secondary outcome, results for each group, and the estimated effect size and its precision (such as 95% confidence interval) | Results |
|  | 17b | For binary outcomes, presentation of both absolute and relative effect sizes is recommended | N/A |
| **Ancillary**  **analyses** | 18 | Results of any other analyses performed, including subgroup analyses and adjusted analyses, distinguishing pre-specified from exploratory | N/A |
| **Harms** | 19 | All important harms or unintended effects in each group (for specific guidance see CONSORT for harms) | N/A |
| **Discussion** | | |  |
| **Limitations** | 20 | Trial limitations, addressing sources of potential bias, imprecision, and, if relevant, multiplicity of analyses | Limitations |
| **Generalisability** | 21 | Generalisability (external validity, applicability) of the trial findings | Discussion |
| **Interpretation** | 22 | Interpretation consistent with results, balancing benefits and harms, and considering other relevant evidence | Discussion |
| **Other information** | | |  |
| **Registration** | 23 | Registration number and name of trial registry | Abstract |
| **Protocol** | 24 | Where the full trial protocol can be accessed, if available | Supporting information |
| **Funding** | 25 | Sources of funding and other support (such as supply of drugs), role of funders | Funding |
