# Supplementary material for: Effects of a WeChat-based PERMA model positive psychological intervention on Chinese women after termination of pregnancy: a randomized controlled trial
Source: Front Psychol. 2026 Jul 20;17:1823918. doi: 10.3389/fpsyg.2026.1823918 (PMC13429383; doi:10.3389/fpsyg.2026.1823918)
Supplement: Supplementary File S3 — Research team roles and responsibilities. [file Table_3.docx]

**Table 1** **Research team roles and responsibilities**

| **Personnel** | **Professional Title** | **Role in Study** | **Responsibilities** | **Independence Statement** |
| --- | --- | --- | --- | --- |
| Director of Nursing Dept | Chief Nurse | Project Leader | Research design & protocol development, coordinating interdisciplinary team collaboration | No involvement in operational execution |
| **Intervention Team** | | | | |
| Head Nurse | Associate Chief Nurse | Intervention Team Member | Participates in designing, adjusting, and guiding intervention implementation | Only aware of intervention group information |
| Nurse A | Associate Chief Nurse | Intervention Team Member | Participates in designing, adjusting, and guiding intervention implementation | Only aware of intervention group information |
| Obstetrician-  Gynecologist A | Attending Physician | Intervention Team Member | Participates in intervention design & implementation; reviews patient eligibility for intervention | Only aware of intervention group information |
| Genetic Counselor | Attending Physician | Intervention Team Member | Participates in intervention design & implementation; provides genetic risk counseling and reproductive planning for post-termination patients | Only aware of intervention group information |
| Psychotherapist | Attending Physician  /Licensed Psychotherapist | Intervention Team Member | Participates in intervention design & implementation;  trains and supervises nurse interventionists;  assesses baseline psychological metrics | Only aware of intervention group information |
| Nurse B (PAC Counselor) | Nurse-in-charge | Intervention Team Member | Participates in intervention implementation; tracks and organizes patient follow-up data | Only aware of intervention group information |
| Nurse C (PAC Counselor) | Staff Nurse | Intervention Team Member | Participates in intervention implementation; tracks and organizes patient follow-up data | Only aware of intervention group information |
| **Control Team** | | | | |
| Nurse D | Nurse-in-charge | Control Team Member | Implementation of routine postpartum care protocols with WeChat group-based consultations and clinical guidance | Only aware of control group information |
| Obstetrician-  Gynecologist B | Attending Physician | Control Team Member | Implementation of routine postpartum care protocols with WeChat group-based consultations and clinical guidance | Only aware of control group information |
| **Support Team** | | | | |
| Nurse E | Nurse-in-charge | Data Manager | Maintains & manages randomized grouping database | Non-blinded, with access to group allocation information |
| Nurse F | Nurse-in-charge | Data Assessor | Conducts data evaluation | Fully blinded and independent from intervention team |
| Nurse G & H | Staff Nurse | Data Statistician | Data entry, organization, and submission to assessors | Fully blinded |
| Nurses I & J | Nursing Intern | Data Collectors | Guide participants in scale completion during the baseline assessment phase | Fully blinded |
