# Supplementary material for: Effects of a WeChat-based PERMA model positive psychological intervention on Chinese women after termination of pregnancy: a randomized controlled trial
Source: Front Psychol. 2026 Jul 20;17:1823918. doi: 10.3389/fpsyg.2026.1823918 (PMC13429383; doi:10.3389/fpsyg.2026.1823918)
Supplement: Supplementary File S4 — Per-protocol analysis of outcome measures. [file Table_4.docx]

**Table.** **Per-protocol (PP) analysis of outcome measures**

| Outcome measure | Group | No. | T0  M ± SD | T1  M ± SD | T2  M ± SD |
| --- | --- | --- | --- | --- | --- |
| Grief (PGS) | Intervention | 50 | 83.35 ± 7.03 | 71.47 ± 5.42 | 67.90 ± 6.92 |
|  | Control | 54 | 83.82 ± 7.60 | 79.04 ± 6.02 | 77.50 ± 5.73 |
|  | t value |  | -1.069 | -7.371 | -7.697 |
|  | p value |  | 0.287 | < 0.001 | < 0.001 |
| PTG (C-PTGI) | Intervention | 50 | 55.18 ± 5.31 | 63.61 ± 8.71 | 68.00 ± 7.93 |
|  | Control | 54 | 56.30 ± 6.18 | 57.52 ± 5.26 | 59.15 ± 4.12 |
|  | t value |  | -1.908 | 4.223 | 7.201 |
|  | p value |  | 0.059 | < 0.001 | < 0.001 |
| SWB (IWB) | Intervention | 50 | 8.98 ± 1.62 | 10.70 ± 2.78 | 11.81± 1.63 |
|  | Control | 54 | 9.12 ± 1.70 | 9.30 ± 1.75 | 9.69 ± 1.12 |
|  | t value |  | -0.305 | 3.127 | 7.777 |
|  | p value |  | 0.761 | 0.002 | < 0.001 |
