# Supplementary material for: Effects of a WeChat-based PERMA model positive psychological intervention on Chinese women after termination of pregnancy: a randomized controlled trial
Source: Front Psychol. 2026 Jul 20;17:1823918. doi: 10.3389/fpsyg.2026.1823918 (PMC13429383; doi:10.3389/fpsyg.2026.1823918)
Supplement: Supplementary File S5 — Detailed intervention protocol. [file Table_5.docx]

Supplementary File S5. Detailed Intervention Protocol

**1 Intervention Team**

1.1 Team composition and roles

The intervention was implemented by a multidisciplinary team. The team composition and specific responsibilities of each member are detailed in Supplementary File S3.

1.2 Provider training

Prior to study commencement, all interventionists completed a standardized training program. The curriculum was designed by the psychotherapist and comprised three modules:

Module 1: Theoretical foundation.

Content covered the PERMA model framework, grief psychology following perinatal loss, cognitive restructuring principles, and cultural sensitivity in communicating with Chinese women who have experienced termination of pregnancy.

Module 2: Technical skills.

This module provided a session-by-session walkthrough of the 8-session protocol, including demonstration of Lamaze breathing guidance, meditation induction, *Baduanjin* (traditional Chinese qigong) instruction, and "Happiness Journal" coaching. Training also covered WeChat group management, including group rule setting, facilitation of online discussions, handling of sensitive disclosures, and management of scheduling conflicts.

Module 3: Simulation and competency assessment.

Each frontline interventionist conducted a mock intervention session with a simulated participant. Performance was evaluated by two independent raters.

Weekly 30-minute debriefing meetings were held throughout the intervention period to review session documentation and address implementation challenges.

**2 Session-by-Session Curriculum (8 Sessions)**

The 4-week intervention comprised eight sessions.

The inpatient phase (Week 1, i.e., Phase 1) included two face-to-face sessions (Sessions 1–2) conducted in a dedicated instructional room within the ward.

The home-based phase (Weeks 2–4, i.e., Phases 2–4) included six remote sessions delivered via the WeChat platform (Sessions 3–8). All sessions followed a standardized four-segment structure:

(1) Mood check-in and homework review (10 minutes);

(2) Didactic content delivery (15 minutes, via mini-lecture slides or pre-recorded video);

(3) Experiential exercise and group discussion (15–20 minutes);

(4) Homework assignment and closing affirmation (5 minutes).

WeChat sessions were scheduled on Wednesday and Friday evenings at 8:00 PM. A reminder message was sent at 10:00 AM on session days to prompt homework review and preparation. Each session lasted approximately 30–50 minutes.

| Week  (Phase) | Theme | Format | Core content and activities |
| --- | --- | --- | --- |
| Week 1 (inpatient) | Recognizing and cultivating positive emotions | Face-to-face | Psychological assessment and emotion release; insight into participants' understanding of their TOP experience and self-identity through narrative sharing; Lamaze breathing exercises (10–20 repetitions); humor-induced positive emotion using a Mr. Bean comedy clip (10 min); introduction to positive psychology principles and the PERMA model. |
| Week 1 (inpatient) | Cultivating positive emotions | Face-to-face | Guided positive cognitive reconstruction; sharing of emotional journeys from diagnosis to hospitalization; highlighting positive perceptions; positive emotion cultivation training (love, curiosity, peace, gratitude, pride, hope); 10-minute tutorial on Happiness Journal completion. |
| Week 2 (home-based) | Enhancing engagement (flow) | WeChat group | Discussion of flow-state experiences in daily proficient activities (housework, reading, exercise, etc.); guided group meditation with soothing background music (15 min). |
| Week 2 (home-based) | Enhancing engagement (flow) | WeChat group | Baduanjin (Eight Pieces of Brocade) exercise video and group practice (15 min); reflection on character strengths and immersive experiences; cultivation of engagement. |
| Week 3 (home-based) | Gratitude and positive relationships | WeChat group | Gratitude dialogue themed "In my story, my child is still alive" (10 min); fertility anxiety counseling; encouragement to discuss fears and aspirations regarding future pregnancy with family, friends, and healthcare providers (20 min). |
| Week 3 (home-based) | Gratitude and positive relationships | WeChat group | Peer experience sharing and social support network building (30 min); active engagement with peers to foster a supportive online community. |
| Week 4 (home-based) | Discovering life meaning | WeChat group | Cognitive reconstruction of the TOP experience and its impact on life; identification of positive growth, shifts in life perspectives and values; support in establishing objectives for health recovery, personal life, and professional development. |
| Week 4 (home-based) | Discovering life meaning and accomplishment | WeChat group | Strengths-based training to enhance self-efficacy; video lecture on happiness philosophy (Prof. Chen Guo, "A Happy Life," 30 min) to encourage acceptance, doing one's best, and embracing what cannot be controlled; drafting of a personalized action plan tailored to current life objectives. |

Homework across phases:

Phase 1: Maintain the Happiness Journal daily, documenting three positive events experienced each day.

Phase 2: Dedicate 15 minutes daily to either meditation or the Baduanjin fitness routine.

Phase 3: Share the personal journey through this experience with a friend or peer, describing the emotional landscape navigated.

Phase 4: Draft an action plan tailored to current recovery and life objectives.

**3 Core Intervention Materials**

3.1 Happiness Journal

Participants received a structured paper booklet titled "Happiness Journal." Each daily page contained three prompts adapted from Seligman's "Three Good Things" exercise:

(1) "Today, one small positive event I experienced is...";

(2) "This event made me feel... (circle: grateful / relieved / proud / hopeful / peaceful / other)";

(3) "I think this happened because..."

Participants were instructed to complete the journal every evening for 28 days, with each entry limited to 50–100 Chinese characters to minimize burden while ensuring reflective depth.

3.2 Weekly lecture PPT

PPTs were sent to the WeChat group prior to each session. Content covered the session theme, core PERMA concepts, and case discussions.

3.3 Educational media and materials

(1) Mr. Bean comedy clip (10 min): Used in Session 1 as a humor-based positive emotion induction to demonstrate that positive emotions can be elicited even during hospitalization.

(2) Guided meditation audio (15 min): Soothing background music with a standardized Chinese-language body-scan script, used in Session 3.

(3) Baduanjin instructional video (15 min): Used in Session 4 as a standardized traditional Chinese qigong exercise video to promote physical engagement, mindful movement, and flow state.

(4) Prof. Chen Guo's lecture video "A Happy Life" (30 min): Used in Session 8 to facilitate meaning reconstruction and life reorientation through philosophical reflection on acceptance and doing one's best.

**4 WeChat Group Management and Family Engagement Rules**

The 59 participants in the intervention group were divided into 6 cohorts based on admission time (5 cohorts with 10 participants each and 1 cohort of 9 participants). Each cohort was established as a closed WeChat group with the following operating rules:

(1) Use of pseudonyms: Participants used self-selected pseudonyms rather than real names to protect privacy.

(2) Asynchronous access: All session materials (slide images, video links, audio files, and written summaries) were permanently retained in the group chat for participants to review at their convenience.

(3) Family involvement: Family members were encouraged to join as "silent supporters" during Weeks 2–4. Their role was limited to observing and encouraging the participant's home practice (e.g., accompanying Baduanjin exercise).

(4) Make-up policy: Participants who missed a live session due to scheduling conflicts (e.g., childcare responsibilities) could review materials asynchronously. For those who missed sessions for other reasons, the intervention team followed up via private message within 24 hours to identify barriers and provide support.

**5 Fidelity Monitoring**

A multi-level fidelity assurance system was implemented to ensure the intervention was delivered as planned and to assess participant adherence:

(1) Process monitoring: During the home-based phase (Weeks 2–4), participants were required to check in via the WeChat group before each session. At the end of each session, a sharing segment invited participants to exchange insights or discuss Happiness Journal entries, serving as evidence of interactive engagement.

(2) Individualized follow-up: For participants who did not check in or engage in interactions, intervention team members followed up individually via private message within 24 hours to identify barriers and provide support.

(3) Attendance incentive: A full-attendance reward mechanism was established, offering participants who completed the entire intervention free follow-up counseling sessions to encourage long-term engagement.
